# Silencing miR‐20a‐5p inhibits axonal growth and neuronal branching and prevents epileptogenesis through RGMa‐RhoA‐mediated synaptic plasticity

**DOI:** 10.1111/jcmm.15677

**Published:** 2020-08-10

**Authors:** Yanyan Feng, Chaojun Duan, Zhaohui Luo, Wenbiao Xiao, Fafa Tian

**Affiliations:** ^1^ Department of Neurology Xiangya Hospital Central South University Changsha China; ^2^ Department of Oncology Xiangya Hospital Central South University Changsha China

**Keywords:** axonal growth, mossy fibre sprouting, neuronal branching, repulsive guidance molecule a, synaptic plasticity

## Abstract

Epileptogenesis is a potential process. Mossy fibre sprouting (MFS) and synaptic plasticity promote epileptogenesis. Overexpression of repulsive guidance molecule a (RGMa) prevents epileptogenesis by inhibiting MFS. However, other aspects underlying the RGMa regulatory process of epileptogenesis have not been elucidated. We studied whether RGMa could be modulated by microRNAs and regulated RhoA in epileptogenesis. Using microRNA databases, we selected four miRNAs as potential candidates. We further experimentally confirmed miR‐20a‐5p as a RGMa upstream regulator. Then, in vitro, by manipulating miR‐20a‐5p and RGMa, we investigated the regulatory relationship between miR‐20a‐5p, RGMa and RhoA, and the effects of this pathway on neuronal morphology. Finally, in the epilepsy animal model, we determined whether the miR‐20a‐5p‐RGMa‐RhoA pathway influenced MFS and synaptic plasticity and then modified epileptogenesis. Our results showed that miR‐20a‐5p regulated RGMa and that RGMa regulated RhoA in vitro. Furthermore, in primary hippocampal neurons, the miR‐20a‐5p‐RGMa‐RhoA pathway regulated axonal growth and neuronal branching; in the PTZ‐induced epilepsy model, silencing miR‐20a‐5p prevented epileptogenesis through RGMa‐RhoA‐mediated synaptic plasticity but did not change MFS. Overall, we concluded that silencing miR‐20a‐5p inhibits axonal growth and neuronal branching and prevents epileptogenesis through RGMa‐RhoA‐mediated synaptic plasticity in the PTZ‐induced epilepsy model, thereby providing a possible strategy to prevent epileptogenesis.

## 1 BACKGROUND

Epilepsy is a common neurological condition that is accompanied by significant morbidity and impacts in excess of 60 million people worldwide.^1^ At present, except for ethosuximide and levetiracetam showing antiepileptogenic properties in a genetic absence epilepsy rat model,^2^ the available antiepileptic drugs only reduce the number of seizures but do not modify the potential pathophysiology. In this regard, there is a need to develop a new disease‐modifying intervention for preventing or treating epilepsy. The mechanisms of epileptogenesis are varied and caused by genetic or acquired factors.^3^ However, the precise mechanisms of epileptogenesis are still unknown. To identify more specific targets for curing epilepsy, we must first understand the mechanisms of epileptogenesis.

Repulsive guidance molecule a (RGMa) was cloned and functionally characterized in 2002^4^. In patients with epilepsy, a microdeletion of the chromosome encompassing the RGMa gene is a potential candidate contributing to epileptogenesis ^5^, ^6^; in animal epilepsy models, growing evidence has shown that RGMa negatively controls epileptogenesis by inhibiting mossy fibre sprouting (MFS).^7^, ^8^, ^9^ In addition, RGMa inhibits synaptic plasticity in the central nervous system ^10^ and synaptic plasticity promotes epileptogenesis.^11^, ^12^ However, whether RGMa is involved in epileptogenesis by regulating synaptic plasticity remains unknown.

In vitro, RGMa inhibits axonal growth and reduces neuronal branching.^13^, ^14^, ^15^, ^16^ RhoA is a key regulator of actin cytoskeletal dynamics.^17^ Similar to RGMa in neurons, functional studies have shown that RhoA inhibits axonal growth and reduces neuronal branching in vitro.^18^, ^19^, ^20^ Importantly, mechanical studies have shown that RGMa suppresses axonal growth by RhoA.^14^, ^21^ The granule cell axon of the hippocampal dentate gyrus is known as mossy fibre. Abnormal granule cell axon development is referred to as MFS. Moreover, changes in neuronal branching affect synaptic structural plasticity. Based on these results, we inferred that through RhoA, RGMa reduces neuronal branching in vitro and suppresses MFS and synaptic plasticity in vivo.

MicroRNAs (miRNAs) exert momentous modulatory effects on gene expression by targeting mRNAs for degradation or translational suppression.^22^ A series of animal and human epilepsy studies have undertaken functional experiments and mechanism analyses of miRNAs,^23^, ^24^ showing that miRNAs represent a powerful mechanism to control protein levels in epilepsy and modify epileptogenesis. As such, miRNAs may be a target to cure epilepsy. Functional studies of miRNAs have suggested that miRNAs regulate axonal growth and neuronal branching in vitro, and MFS and synaptic plasticity in vivo.^25^, ^26^, ^27^, ^28^, ^29^ Hence, we conjectured that miRNAs act as key upstream molecules of RGMa in regulating axonal growth and neuronal branching in vitro, and MFS and synaptic plasticity in epileptogenesis.

In summary, we supposed that miRNAs regulate RGMa and that RhoA is a downstream factor of RGMa in vitro. Based on this hypothesis, we further inferred that the miRNAs‐RGMa‐RhoA pathway regulates axonal growth and neuronal branching in vitro, and MFS and synaptic plasticity in epileptogenesis. According to our results, miR‐20a‐5p regulated RGMa, and RGMa regulated RhoA in PC12 cells and primary hippocampal neurons. Additionally, the miR‐20a‐5p‐RGMa‐RhoA pathway regulated neuronal morphology in primary hippocampal neurons. RGMa suppressed synaptic plasticity in epileptogenesis, apart from inhibiting MFS. Finally, silencing miR‐20a‐5p prevented epileptogenesis via RGMa‐RhoA‐mediated synaptic plasticity but did not change MFS in a pentylenetetrazol (PTZ)‐induced epilepsy model.

## 2 MATERIALS AND METHODS

### 2.1 In silico prediction

Three open miRNA databases, miRDB (http://mirdb.org/), Targetscan 7.2 (http://www.targetscan.org/vert_72/) and DIANA tools microT‐CDS (http://diana.imis.athena‐innovation.gr/DianaTools/index.php?r=microT_CDS/index), were employed to identify possible binding miRNAs of the RGMa 3′ untranslated region (3′UTR). miRNAs identified by all three tools were analysed, and four miRNAs with relatively high scores in all three databases were selected.

### 2.2 Animals

Adult (male, weight: 200‐220 g, age: 7‐9 weeks old) and post‐natal day 1 (male and female) Sprague‐Dawley rats were acquired from the Central South University Animal Center. The rats were given ad libitum access to food and water under controlled lighting and temperature conditions (12‐hours light/dark cycle, 22‐25°C). All animal protocols were conducted according to institutional guidelines and were authorized by the ethics committee of Xiangya Hospital, Central South University.

In this study, we randomly divided adult rats into six groups. The rats in the normal control (NC) group received only 0.9% NaCl saline intraperitoneally (ip). The rats in the PTZ group received only PTZ (30 mg/kg, ip). Rats assigned to the anti‐RGMa + PTZ and IgG + PTZ groups were pre‐treated with the functional RGMa antibody (28045, IBL) or IgG (I5006, Sigma) (intracerebroventricular injection), respectively, and then administered PTZ (30 mg/kg, ip) 24 hours later. Six days after PTZ administration, the animals were killed. Rats in the Sp‐NC + PTZ and Sp‐miR‐20a + PTZ groups were pre‐treated with adeno‐associated virus 9 (AAV9) vectors expressing ZsGreen1 (Sp‐NC) or AAV9 vectors expressing miR‐20a sponge and ZsGreen1 (Sp‐miR‐20a) (Hanbio Biotechnology, China, intradentate gyrus administration), respectively, and then injected with PTZ (30 mg/kg, ip) four weeks later. The rats in the Sp‐NC + PTZ and Sp‐miR‐20a + PTZ groups were randomly divided into two groups according to the first injection time of PTZ: 3 and 14 days.

### 2.3 Cell culture

293T cells, PC12 cells and primary hippocampal neurons were used in the present study. Details were listed in Methods S1.

### 2.4 Transfections

An miR‐20a‐5p inhibitor, an miR‐106b‐5p inhibitor, an inhibitor NC, a small interfering RNA (siRNA) targeting RGMa (si‐RGMa) and a control siRNA (si‐Ctrl) were synthesized by Sangon Biotech (China) and transfected into PC12 cells with Lipofectamine 2000 (Invitrogen, USA) according to the recommended procedures. Lentivirus vectors expressing pre‐miR‐20a (pre‐miR‐20a) and miR‐20a sponge (Sp‐miR‐20a) sequences were purchased from GeneChem (China) and Vigene (China), respectively. Lentiviral vectors carrying enhanced green fluorescent protein (pre‐NC) or green fluorescent protein (Sp‐NC) served as the corresponding controls. By screening for puromycin‐resistant cells, we established PC12 cell lines stably expressing pre‐miR‐20a and miR‐20a sponge. A functional RGMa antibody/IgG (10 µg/mL) was used to interfere with RGMa function in vitro.

To disturb RGMa function in vivo, we bilaterally administered intracerebroventricular injections of 5 µg of the functional RGMa antibody/IgG to rats at stereotactic coordinates from bregma [(anterior‐posterior, medial‐lateral and dorsal‐ventral (subdural)] (in mm): −0.8, −1.0, 3.4 and −0.8, +1.0, 3.4.

To silence miR‐20a‐5p in vivo, we bilaterally injected 2.0 × 10^9^ viral genomes of AAV‐mediated Sp‐miR‐20a or Sp‐NC vectors into the dentate gyri of rats located 2.5 mm posterior from bregma, 1.0 mm lateral (on each side) from the midline and 5.0 mm deep from the skull surface at a 0.2 mL/min rate. The vectors contained an hSyn promoter to guide specific expression in neurons of the dentate gyrus (DG).

### 2.5 Behaviour analysis

Seizure responses were observed over 2 hours following PTZ injection every day and classified according to previous studies by a modified description of the Racine score (1972) as follows: 0‐no responses, 1‐mouth and facial cramps, 2‐head nodding, 3‐forelimb clonus, 4‐rearing, and 5‐rearing and falling. The animals were injected with PTZ until they reached the successful kindling criterion: class 3, 4 or 5 seizures exhibited five times during five consecutive injections.

### 2.6 Sample collection for RT‐qPCR/ Western blot and RNA/protein isolation

Details were listed in Methods S1.

### 2.7 Luciferase/Renilla assay

Luciferase/Renilla assay to evaluate the direct binding of miR‐20a‐5p to the RGMa 3′UTR.

Details were listed in Methods S1.

### 2.8 RT‐qPCR

RT‐qPCR to assess miRNA and RGMa mRNA expression changes and **Western blot** and **Immunofluorescence** staining to assess protein expression.

Details were listed in Methods S1.

### 2.9 Axonal length measurement and Sholl analysis

Details were listed in Methods S1.

### 2.10 Slice preparation

To perform fluorescent scanning, Timm staining and immunofluorescence staining, we collected eight rat brains each from the IgG + PTZ and anti‐RGMa + PTZ groups, four rat brains each from the NC and PTZ groups and eight rat brains each from the Sp‐NC + PTZ and Sp‐miR‐20a + PTZ groups at 3 and 14 days via a transcardial perfusion procedure.

Details were listed in Methods S1.

### 2.11 Fluorescent scanning

To determine the sites infected with Sp‐miR‐20a and Sp‐NC vectors in the DG region, we observed the expression of ZsGreen1 in the brain. Three random slides from rats in the Sp‐NC + PTZ and Sp‐miR‐20a + PTZ groups were scanned with a CaseViewer 2.0 microscope (3DHISTECH, Hungary) at each time‐point. Slices were dyed with Hoechst 33342 before scanning.

### 2.12 Timm staining to assess MFS

Details were listed in Methods S1.

### 2.13 Statistical analysis

Data are expressed as the mean and standard deviation or the median and interquartile range. The Shapiro‐Wilk test and Kolmogorov‐Smirnov test were used to evaluate the normal distribution of the data, and the Levene test was used to compare variances before we used a parametric test to compare the differences between groups. If data did not fulfil the requirements of a parametric test, a non‐parametric test would be selected. We used the independent sample t test, Mann‐Whitney test, analysis of variance (ANOVA), Kruskal‐Wallis test and a log linear model to determine the differences of the data. Tukey, Sidak, Nemenyi and Dunnett tests were used for multiple comparisons. The results were considered statistically significant when *P* < 0.05. All statistical analyses were two‐sided and were conducted using the Statistical Package for the Social Sciences version 23.0 and GraphPad Prism 8. Illustrator CC and Photoshop CC were used to make graphs.

## 3 RESULTS

### 3.1 miR‐20a‐5p is a candidate regulator of RGMa expression in a PTZ‐induced epilepsy rat model

**FIGURE 1:**
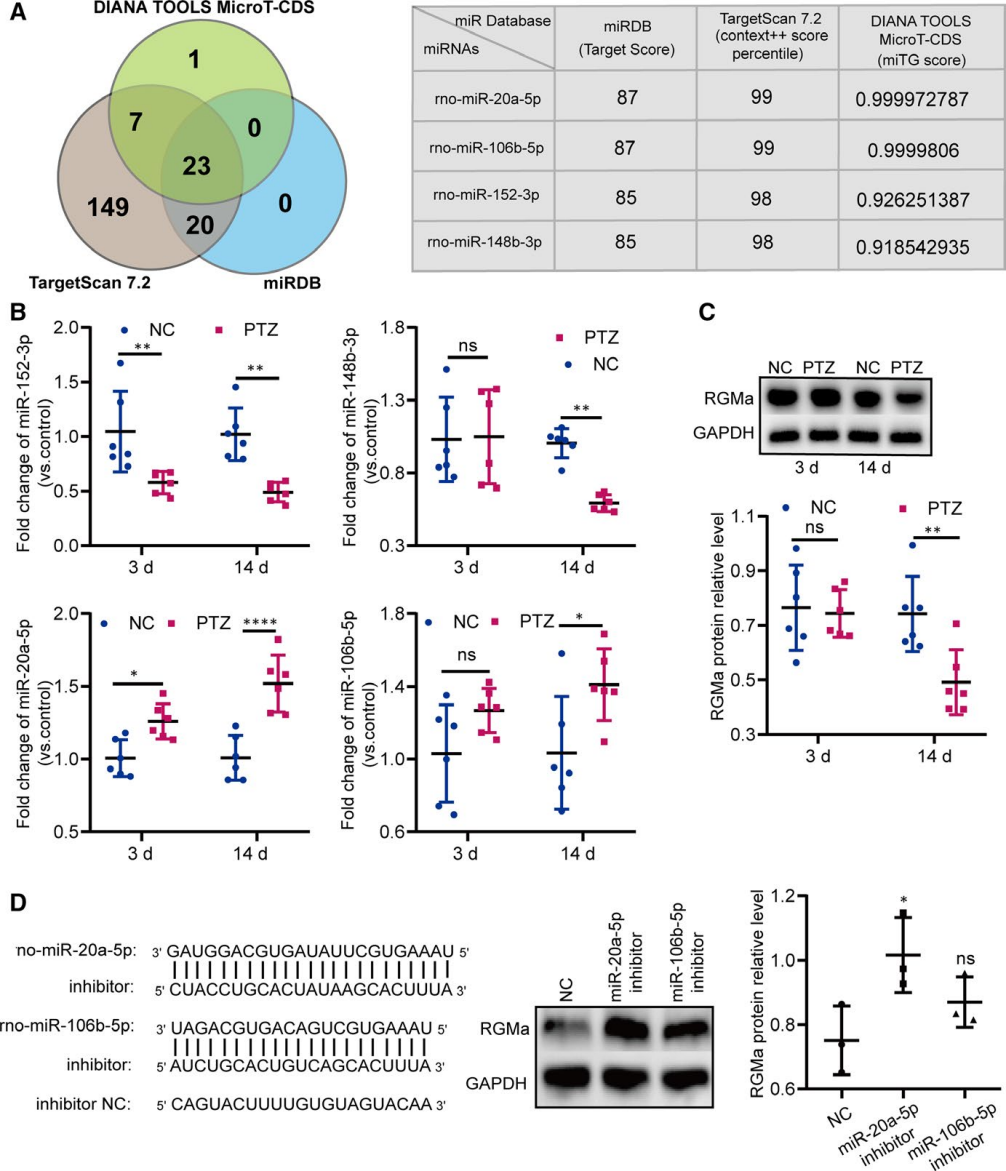
Identifying miR‐20a‐5p as an upstream regulator of RGMa in a PTZ‐induced epilepsy model. A, In silico prediction of 3'UTR‐RGMa binding sites. The Venn diagram shows the number of miRNAs predicted by different algorithms. Twenty‐three candidates were identified by all three tools (DIANA TOOLS MicroT‐CDS, TargetScan 7.2 and miRDB). Among these twenty‐three candidates, the table depicts the scores of four (miR‐106b‐5p, miR‐148b‐3p, miR‐152‐3p and miR‐20a‐5p), all with relatively high scores in all three databases. B, RT‐qPCR results revealing the differences in the expression levels of the four miRNAs (miR‐106b‐5p, miR‐148b‐3p, miR‐152‐3p and miR‐20a‐5p) in PTZ‐induced epilepsy rats at 3 and 14 d (n = 6; two‐way ANOVA, Sidak test). C, Western blot results revealing RGMa protein expression in PTZ‐induced epilepsy rats at 3 and 14 d (n = 6; two‐way ANOVA, Sidak test). D, Based on the observation that miRNAs often negatively regulate the target gene combined with the results in B and C, we designed miR‐20a‐5p and miR‐106b‐5p inhibitors to suppress their expression in PC12 cells. Western blot results revealed that, compared with NC, miR‐20a‐5p inhibitors distinctly reduced RGMa protein expression in PC12 cells. Furthermore, miR‐20a‐5p inhibitors more powerfully regulated RGMa protein expression than miR‐106b‐5p inhibitors (n = 3 independent cell culture preparations; one‐way ANOVA, Dunnett test) (^ns^ *P* ≥ 0.05, *P < 0.05, **P < 0.01, ****P < 0.0001). All data represent the mean ± standard deviation (SD)

Emerging evidence has indicated that RGMa inhibits MFS and epileptogenesis.^7^, ^8^, ^9^ To identify the upstream regulators of RGMa, we performed a non‐biased search of in silico databases and intersected this information with publicly available miRNA profiles that potentially bind the 3'UTR of RGMa. Among the twenty‐three miRNAs identified in all three databases, four miRNAs with relatively higher scores, including miR‐106b‐5p, miR‐148b‐3p, miR‐152‐3p and miR‐20a‐5p, were selected as potential candidates (Figure 1A) and their levels were measured in rats of the NC and PTZ groups by RT‐qPCR at 3 and 14 days. Compared with the levels in the NC group, hippocampal miR‐148b‐3p and miR‐152‐3p were down‐regulated, while miR‐106b‐5p and miR‐20a‐5p were significantly up‐regulated in the PTZ group (Figure 1B). Rats in the PTZ group expressed lower RGMa protein levels than those in the NC group (Figure 1C). Based on the negative relationship between miRNAs and their downstream targets, we designated miR‐20a‐5p and miR‐106b‐5p as potential upstream regulators of RGMa. Next, we employed miRNA inhibitors to decrease miR‐20a‐5p and miR‐106b‐5p expression in PC12 cells. Data showed that the expression of RGMa protein was significantly increased by the miR‐20a‐5p inhibitor (Figure 1D). Therefore, we selected miR‐20a‐5p as a candidate regulator of RGMa expression in a PTZ‐induced epilepsy model.

### 3.2 miR‐20a‐5p targets the 3'UTR‐RGMa sequence to regulate RGMa expression

**FIGURE 2:**
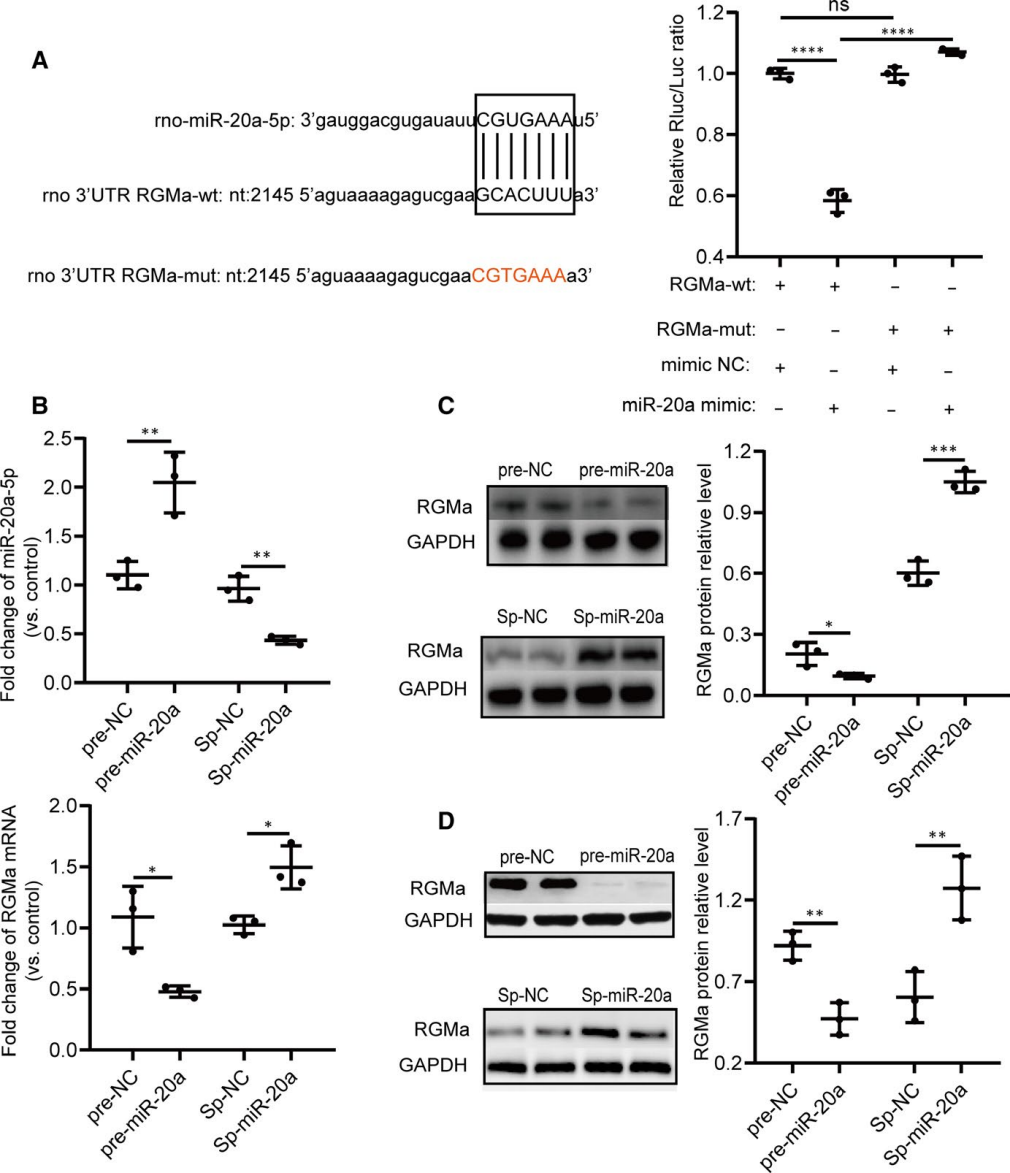
miR‐20a‐5p targets the 3'UTR RGMa sequence, and manipulation of miR‐20‐5p regulates RGMa expression in vitro. A, miR‐20a‐5p binds to the 3'UTR of RGMa. The miR‐20a‐5p target region on the rat RGMa 3'UTR and the seed region are shown in capital letters. miR‐20a‐5p mimics significantly down‐regulated luciferase activity compared with the control (RGMa‐wt + NC vs RGMa‐wt + mimic). The mutated sequence of the RGMa 3'UTR, shown in red, abolished the suppressive effect of miR‐20a‐5p mimics and augmented luciferase activity in comparison with the wild‐type reporter (RGMa‐wt + mimic vs RGMa‐mut + mimic). In the absence of miR‐20a‐5p mimics, the mutated sequence of the RGMa 3'UTR had no significant influence on luciferase activity (RGMa‐wt + NC vs RGMa‐mut + NC). (one‐way ANOVA, Tukey test). B, Pre‐miRNA is processed into mature miRNAs and increases the biological activity of the miRNA. A miRNA sponge absorbs miRNAs and suppresses their function. The expression of miR‐20a‐5p was higher in the pre‐miR‐20a group and lower in the Sp‐miR‐20a group than in the corresponding control groups in PC12 cells. The RGMa mRNA fold change (vs control) was less than one in the pre‐miR‐20a group and greater than one in the Sp‐miR‐20a group in PC12 cells. (two‐tailed t test). C, The RGMa protein fold change was less than one in the pre‐miR‐20a group and greater than one in the Sp‐miR‐20a group in PC12 cells (two‐tailed t test). D, Corresponding to the modulation of miR‐20a‐5p expression in PC12 cells, RGMa protein expression was lower in the pre‐miR‐20a group and higher in the Sp‐miR‐20a group than in the corresponding control groups in primary hippocampal neurons (two‐tailed t test). (^ns^ *P* ≥ 0.05, *P < 0.05, **P < 0.01, ***P < 0.001, ****P < 0.0001; n = 3 independent cell culture preparations). All data represent the mean ± SD

Next, we used a luciferase assay to determine whether miR‐20a‐5p binds to the 3'UTR of RGMa. The 3'UTR of RGMa was mutated; the mutated sequence is shown in Figure 2A. Compared with that in the mimic NC + RGMa‐wt group, the luciferase activity in the miR‐20a mimic + RGMa‐wt group was obviously decreased, while compared with that in the miR‐20a mimic + RGMa‐wt group, the luciferase activity in the miR‐20a mimic + RGMa‐mut group was significantly increased (Figure 2A), indicating that miR‐20a‐5p targets the 3'UTR‐RGMa sequence. The functional relationship of miR‐20a‐5p and RGMa was then explored in depth. Pre‐miR‐20a and miR‐20a sponge were used to manipulate miR‐20a‐5p expression, and RT‐qPCR revealed that miR‐20a‐5p expression was controlled in PC12 cells (Figure 2B). Meanwhile, compared with the corresponding control groups of PC12 cells, the pre‐miR‐20a group expressed lower RGMa mRNA and protein levels, whereas the Sp‐miR‐20a group expressed higher levels (Figure 2B,C). Moreover, modulation of miR‐20a‐5p was performed in primary hippocampal neurons, and the RGMa protein was detected by Western blot. The RGMa protein level was obviously higher in the Sp‐miR‐20a group and lower in the pre‐miR‐20a group than in the corresponding control groups in primary hippocampal neurons (Figure 2D). Collectively, these data suggested that manipulation of miR‐20a‐5p regulates RGMa expression.

### 3.3 Silencing miR‐20a‐5p inhibits axonal growth and neuronal branching in primary hippocampal neurons by the RGMa‐RhoA pathway

**FIGURE 3:**
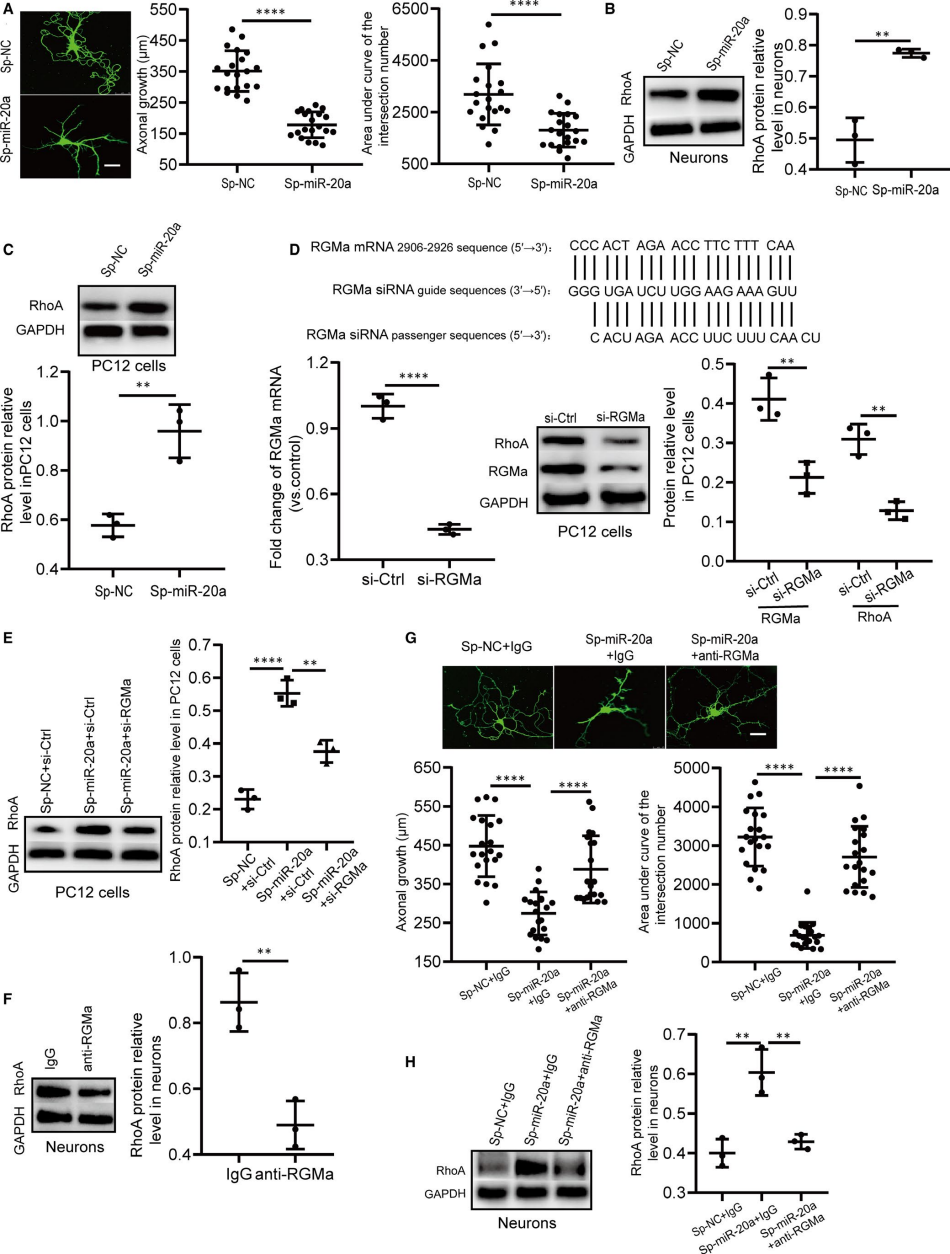
Silencing miR‐20a‐5p inhibits primary hippocampal neuronal branching and axonal growth by regulating the RGMa‐RhoA pathway. A, Representative primary hippocampal neurons in the Sp‐miR‐20a and Sp‐NC groups captured by confocal laser scanning microscopy (bar = 25 µm). The axonal lengths of primary hippocampal neurons in Sp‐miR‐20a rats were significantly shortened compared with those in Sp‐NC rats (n = 20; two‐tailed t test). Neuronal morphology was assessed by Sholl analysis. Compared with that in the Sp‐NC group, the area under curve of the intersection numbers in the Sp‐miR‐20a group was significantly reduced (n = 20; two‐tailed t test), suggesting that silencing miR‐20a‐5p reduces neuronal branching of primary hippocampal neurons. B, miR‐20a sponge obviously decreased RhoA levels in primary hippocampal neurons (n = 3 independent cell culture preparations; two‐tailed t test). C, Down‐regulation of miR‐20a‐5p using a miRNA sponge distinctly increased RhoA expression in PC12 cells (n = 3 independent cell culture preparations; two‐tailed t test). D, RGMa‐siRNA targeting sites and sequences. RGMa mRNA and protein levels were down‐regulated by RGMa‐siRNA in PC12 cells (n = 3 independent cell culture preparations; two‐tailed t test). When the level of RGMa was decreased, the RhoA protein level was also decreased in PC12 cells (n = 3 independent cell culture preparations, two‐tailed t test). E, RGMa‐siRNA reversed the increase in RhoA induced by miR‐20a‐5p in PC12 cells (n = 3 independent cell culture preparations; one‐way ANOVA, Dunnett test). F, The functional RGMa antibody down‐regulated RhoA expression in primary hippocampal neurons (n = 3 independent cell culture preparations; two‐tailed t test). G, Representative primary hippocampal neurons in the Sp‐NC + IgG, Sp‐miR‐20a + IgG and Sp‐miR‐20a + anti‐RGMa groups captured by confocal laser scanning microscopy (bar = 25 µm). Compared with that in the Sp‐NC + IgG group, axonal growth in the Sp‐miR‐20a + IgG group was significantly shortened. When the functional antibodies were added to primary hippocampal neurons infected by miR‐20a sponge vectors, axonal growth was extended (n = 20; one‐way ANOVA, Dunnett test). Similar to the effects of the functional RGMa antibody on axonal growth, the functional RGMa antibody had the same influence on the area under the curve of the intersection numbers (n = 20; one‐way ANOVA, Dunnett test). H, The functional RGMa antibody reversed the up‐regulating effect of miR‐20a sponge on RhoA expression in primary hippocampal neurons (n = 3 independent cell culture preparations; one‐way ANOVA, Dunnett test). (**P < 0.01, ****P < 0.0001). All data represent the mean ± SD

Past studies have indicated that RGMa inhibits axonal growth and reduces neuronal branching.^14^, ^15^ Based on miR‐20a‐5p as an upstream regulator of RGMa, we inferred that miR‐20a‐5p controls axonal growth and neuronal branching. To determine the influences of miR‐20a‐5p on axonal growth and neuronal branching, we measured the axonal lengths of primary hippocampal neurons and assessed neuronal branching when primary hippocampal neurons were infected with a sponge of miR‐20a. The results showed that silencing miR‐20a‐5p inhibited axonal growth and neuronal branching in primary hippocampal neurons (Figure 3A).

miR‐20a‐5p induces the axonal growth of primary sensory neurons via RhoA.^30^ Additionally, RGMa inhibits axonal growth by suppressing RhoA.^14^ Therefore, miR‐20a‐5p may exert its function through the RGMa‐RhoA pathway, as supposed. When miR‐20a‐5p was down‐regulated by a sponge of miR‐20a in primary hippocampal neurons and PC12 cells, the level of RhoA was significantly increased (Figure 3B,C). To explore whether RGMa reverses the effect of miR‐20a‐5p on RhoA in PC12 cells, we designed the RGMa‐siRNA sequence to degrade RGMa mRNA (Figure 3D). At the mRNA level, the amount of RGMa mRNA was decreased by RGMa‐siRNA by more than 50%; at the protein level, RGMa protein expression was also strikingly decreased. When the level of RGMa was decreased, a reduction in RhoA protein was also detected (Figure 3D). We then transfected RGMa‐siRNA or its control into PC12 cells stably expressing miR‐20a sponge or its corresponding control and measured the expression levels of RGMa and RhoA. The elevation in RhoA expression was significantly reversed by RGMa‐siRNA (Figure 3E). These results suggested that RGMa successfully reverses the influence of miR‐20a‐5p on RhoA in PC12 cells.

RhoA not only inhibits axonal growth but also regulates neuronal branching.^31^ Next, we validated whether decreased RGMa rescued the influences of miR‐20a‐5p down‐regulation on RhoA and neuronal morphology in primary hippocampal neurons. A functional RGMa antibody was used to disturb RGMa function in primary hippocampal neurons. Compared with that in the IgG group, the level of RhoA in the anti‐RGMa group was markedly decreased (Figure 3F), indicating that the functional RGMa antibody interferes with the RGMa function of primary hippocampal neurons. Furthermore, we determined whether decreased RGMa rescued the influences of silencing miR‐20a‐5p on the axonal growth and neuronal branching of primary hippocampal neurons. Compared with that in the Sp‐NC + IgG group, the axonal growth in the Sp‐miR‐20a + IgG group was significantly shortened and the area under curve of the intersection numbers in the Sp‐miR‐20a + IgG group was decreased. When functional RGMa antibodies were added to primary hippocampal neurons infected by miR‐20a sponge vectors, the axonal growth was extended and the area under curve of the intersection numbers was increased (Figure 3G). These data suggested that the functional RGMa antibody successfully rescues the inhibitory effects of the miR‐20a‐5p sponge on axonal growth and neuronal branching. Meanwhile, the level of RhoA in the Sp‐miR‐20a + anti‐RGMa group was lower than that in the Sp‐miR‐20a + IgG group (Figure 3H). Taken together, these findings suggested that silencing miR‐20a‐5p inhibits axonal growth and neuronal branching in primary hippocampal neurons via the RGMa‐RhoA pathway.

### 3.4 RGMa inhibits MFS and synaptic plasticity in epileptogenesis

**FIGURE 4:**
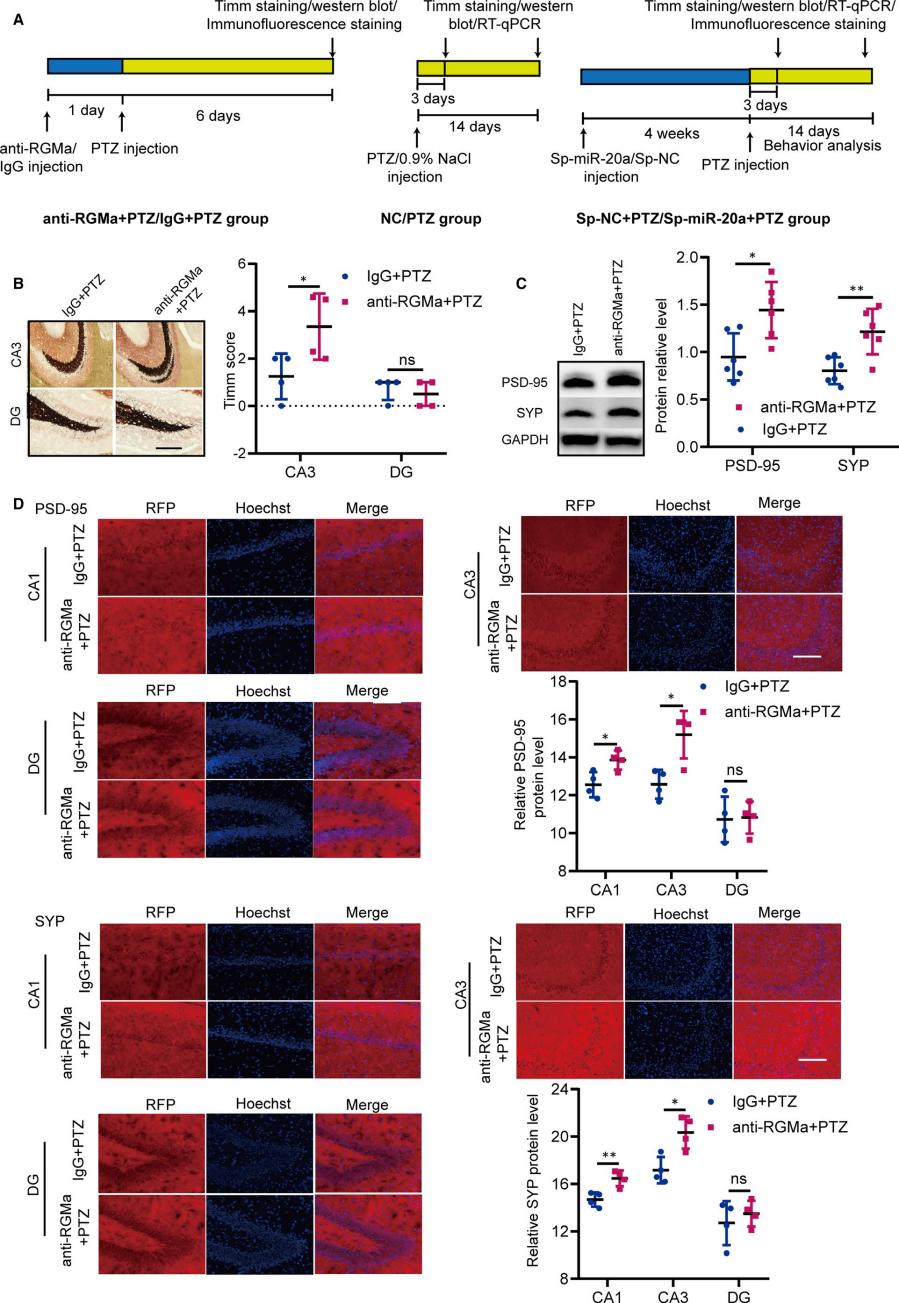
The functional RGMa antibody increases MFS and synaptic plasticity in a PTZ‐induced epilepsy model. A, The schemes show the in vivo experimental design. B, Representative pictures of MFS in the CA3 and DG regions of rats in the IgG + PTZ and anti‐RGMa + PTZ groups (bar = 200 µm). Compared with that in IgG + PTZ rats, the MFS of the CA3 region in anti‐RGMa + PTZ rats differed significantly (n = 4; two‐tailed t test; data represent the mean ± SD). MFS in the DG region was not significantly different between groups (n = 4; Mann‐Whitney test; data represent the median ± interquartile range). C, The protein levels of PSD‐95 and SYP in the IgG + PTZ and anti‐RGMa + PTZ groups were detected by Western blot. PSD‐95 and SYP protein levels were higher in anti‐RGMa + PTZ rats than in IgG + PTZ rats (n = 6; two‐tailed t test; data represent the mean ± SD). D, The levels of PSD‐95 and SYP were detected by immunofluorescence. The levels of PSD‐95 and SYP in the CA1 and CA3 regions of anti‐RGMa + PTZ rats were higher than those in the CA1 and CA3 regions of control rats. Their expression levels in the DG region were not obviously different between groups (n = 4; two‐tailed t test; data represent the mean ± SD). (^ns^ *P* ≥ 0.05, *P < 0.05, **P < 0.01)

Our above data indicated that the miR‐20a‐5p‐RGMa‐RhoA pathway regulates axonal growth and neuronal branching in vitro. Based on the in vitro results, we confirmed that RGMa suppressed synaptic plasticity in a PTZ‐induced epilepsy model, except for inhibiting MFS in epileptogenesis. The in vivo experimental designs are shown in Figure 4A. When the functional RGMa antibody was injected into rats, MFS was significantly up‐regulated in anti‐RGMa + PTZ rats compared with that in IgG + PTZ rats (Figure 4B). We measured the levels of the synaptic proteins PSD‐95 and SYP to indirectly evaluate synaptic plasticity.^32^ Western blot analysis revealed that PSD‐95 and SYP expression levels were higher in anti‐RGMa + PTZ rats than in IgG + PTZ rats (Figure 4C). We then used immunofluorescence staining to detect the levels of PSD‐95 and SYP in the CA1, CA3 and DG regions of the hippocampus. The levels of PSD‐95 and SYP were higher in the CA1 and CA3 regions of anti‐RGMa + PTZ rats than in those of IgG + PTZ rats, but the two groups did not exhibit significant differences in PSD‐95 or SYP immunoreactivity in the DG region (Figure 4D). These data indicated that RGMa inhibits not only MFS but also synaptic plasticity in PTZ‐induced epilepsy rats.

### 3.5 miR‐20a‐5p down‐regulation suppresses synaptic plasticity in epileptogenesis

**FIGURE 5:**
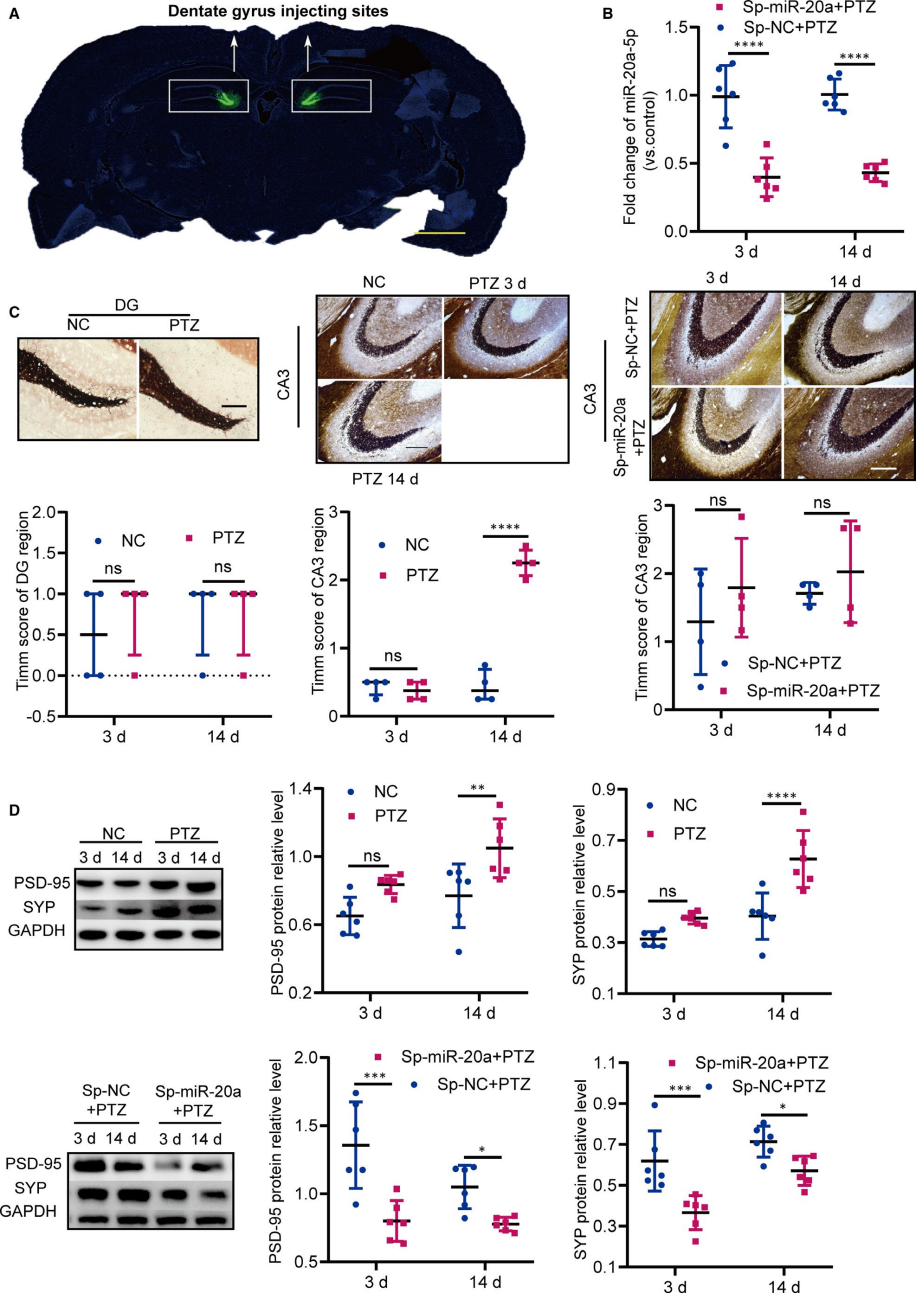
Silencing miR‐20a‐5p decreases synaptic plasticity in a PTZ‐induced epilepsy model. A, miRNA sponge‐expressing adeno‐associated viral particles silenced miR‐20a‐5p in the dentate gyri of rats. ZsGreen1 is a flag tag. Representative brain slices show the DG injection sites (bar = 2000 µm). B, The level of miR‐20a‐5p in Sp‐miR‐20a + PTZ rats was distinctly down‐regulated compared with the level in control rats (n = 6, two‐way ANOVA, Sidak test; data represent the mean ± SD). C, Representative pictures of MFS in the CA3 and DG regions (bar = 200 µm). No significant MFS was observed in the NC and PTZ groups (n = 4, Kruskal‐Wallis test, Nemenyi test; data represent the median ± interquartile range). MFS of the hippocampal CA3 region in PTZ‐induced epilepsy rats was distinctly increased during the kindling process in comparison with that in NC rats at each time‐point (n = 4, Kruskal‐Wallis test, Nemenyi test; data represent the median ± interquartile range). Silencing miR‐20a‐5p did not induce changes in MFS in the hippocampal CA3 region in Sp‐miR‐20a + PTZ rats at either time‐point (n = 4, two‐way ANOVA, Sidak test; data represent the mean ± SD). D, The levels of PSD‐95 and SYP were detected in the NC and PTZ groups by Western blot, revealing that PSD‐95 and SYP protein levels were higher in PTZ‐induced epilepsy rats than in control rats at 14 d. When the expression of miR‐20a was silenced, compared with the levels in the Sp‐NC + PTZ group, the levels of PSD‐95 and SYP in the Sp‐miR‐20a + PTZ group were significantly decreased (n = 6, two‐way ANOVA, Sidak test; data represent the mean ± SD). (^ns^ *P* ≥ 0.05, *P < 0.05, **P < 0.01, ***P < 0.001, ****P < 0.0001)

Next, the biological functions of miR‐20a‐5p in vivo were determined by silencing using an AAV delivery system. The in vivo experimental designs are shown in Figure 4A. We attempted to deplete miR‐20a‐5p in the dentate gyri of rats using a sponge of miR‐20a. Prior to confirming the roles of the sponge, we verified that DG neurons were infected by the miR‐20a sponge (Figure 5A). Then, the level of miR‐20a‐5p in the hippocampal tissues at the injection sites was measured by RT‐qPCR. Compared with the level of miR‐20a‐5p in control rats, its level was strongly decreased in Sp‐miR‐20a + PTZ rats at each time‐point (Figure 5B), suggesting that the miR‐20a sponge silences miR‐20a‐5p expression in the PTZ‐induced epilepsy model.

Then, we determined whether silencing miR‐20a‐5p reversed MFS in PTZ‐induced rats. MFS in the CA3 and DG regions was detected in the NC and PTZ groups at 3 and 14 days. We did not observe distinct MFS differences in the DG between groups at either time‐point (Figure 5C). The MFS of the hippocampal CA3 region was significantly higher during the kindling process in rats of the PTZ group than in control rats (Figure 5C). To further determine the roles of miR‐20a‐5p in MFS, we evaluated MFS in the hippocampal CA3 region in the setting of reduced miR‐20a‐5p expression at 3 and 14 days. In comparison with MFS in the hippocampal CA3 region of control animals, MFS in that of Sp‐miR‐20a + PTZ rats was not reversed at either time‐point (Figure 5C). These data demonstrated that decline in miR‐20a‐5p expression does not reverse MFS in the hippocampal CA3 region of PTZ‐induced epilepsy model rats.

**FIGURE 6:**
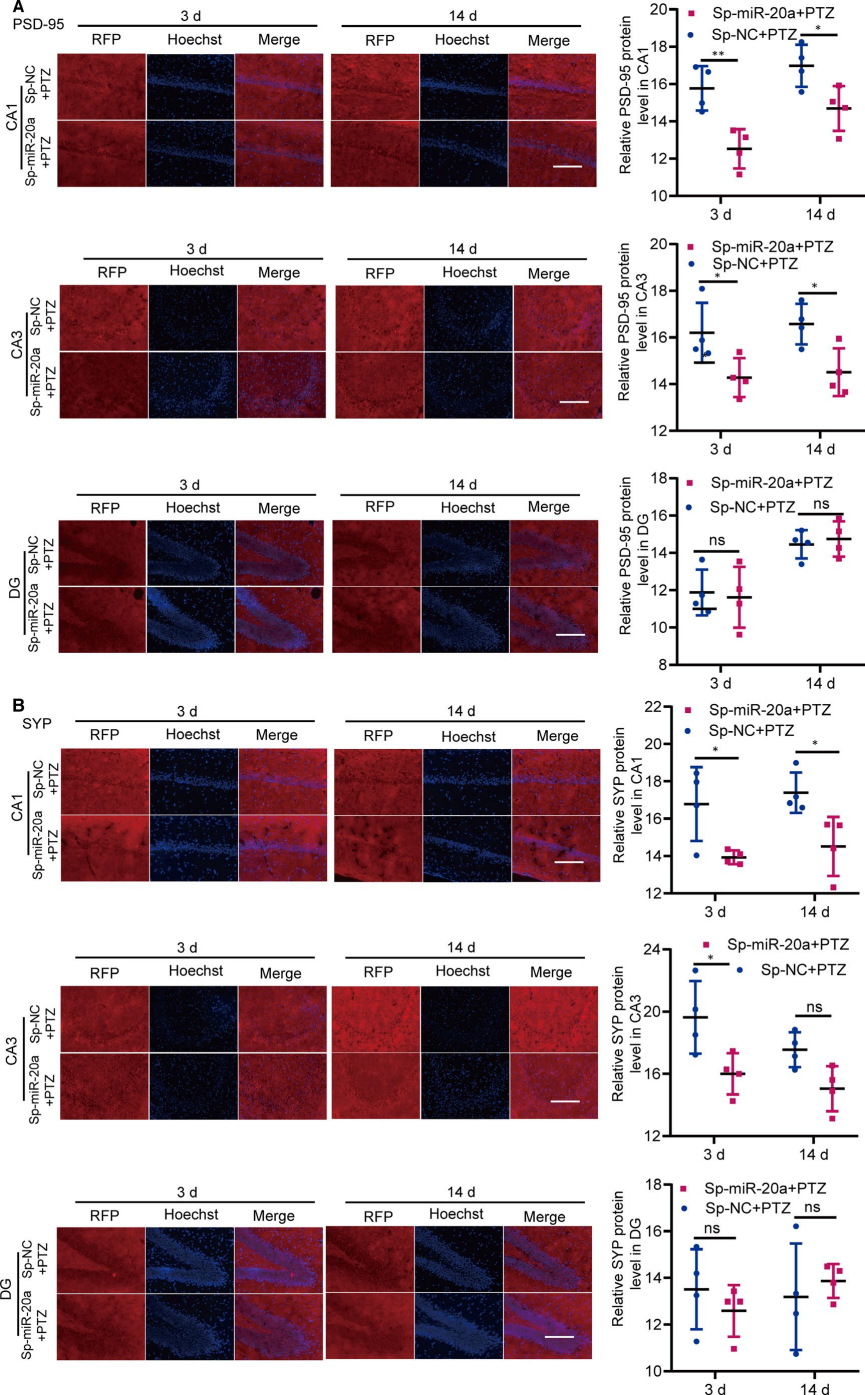
The PSD‐95 (A) and SYP (B) levels in the Sp‐NC + PTZ and Sp‐miR‐20a + PTZ groups were measured by immunofluorescence. Representative fluorescence pictures of the CA3, CA1 and DG regions are displayed (bar = 150 µm). The levels of PSD‐95 and SYP in the CA3 region were significantly decreased in the Sp‐miR‐20a + PTZ group compared with those in the Sp‐NC + PTZ group at 3 d. At 14 d, compared with the Sp‐NC + PTZ group, the expression of PSD‐95 in the CA3 region was significantly decreased in the Sp‐miR‐20a + PTZ group, while the level of SYP in the CA3 region was not significantly difference from that of the Sp‐miR‐20a + PTZ group. The PSD‐95 and SYP levels in the DG region were not distinctly different between groups at 3 d or 14 d. (n = 4, ^ns^ *P* ≥ 0.05, *P < 0.05, **P < 0.01; two‐way ANOVA, Sidak test). All data represent the mean ± SD

Finally, we determined whether decreased miR‐20a‐5p altered synaptic plasticity in PTZ‐induced epilepsy rats. We tested the hippocampal levels of PSD‐95 and SYP by Western blot in the NC and PTZ groups at 3 and 14 days. The hippocampal levels of PSD‐95 and SYP were obviously higher in PTZ rats than in NC rats at 14 days (Figure 5D). When miR‐20a‐5p was down‐regulated, Western blot analysis demonstrated that the levels of PSD‐95 and SYP in injection sites were significantly lower in Sp‐miR‐20a + PTZ rats than in control rats at 3 and 14 days (Figure 5D); immunofluorescence analysis demonstrated that the levels of PSD‐95 and SYP were down‐regulated in the CA1 and CA3 regions of the hippocampus at each time‐point (Figure 6). These results indicated that silencing miR‐20a‐5p varies synaptic plasticity in the PTZ‐induced epilepsy model.

### 3.6 Antagonizing miR‐20a‐5p prevents epileptogenesis, and the miR‐20a‐5p‐RGMa‐RhoA pathway is involved in this process in the PTZ‐induced epilepsy model

**FIGURE 7:**
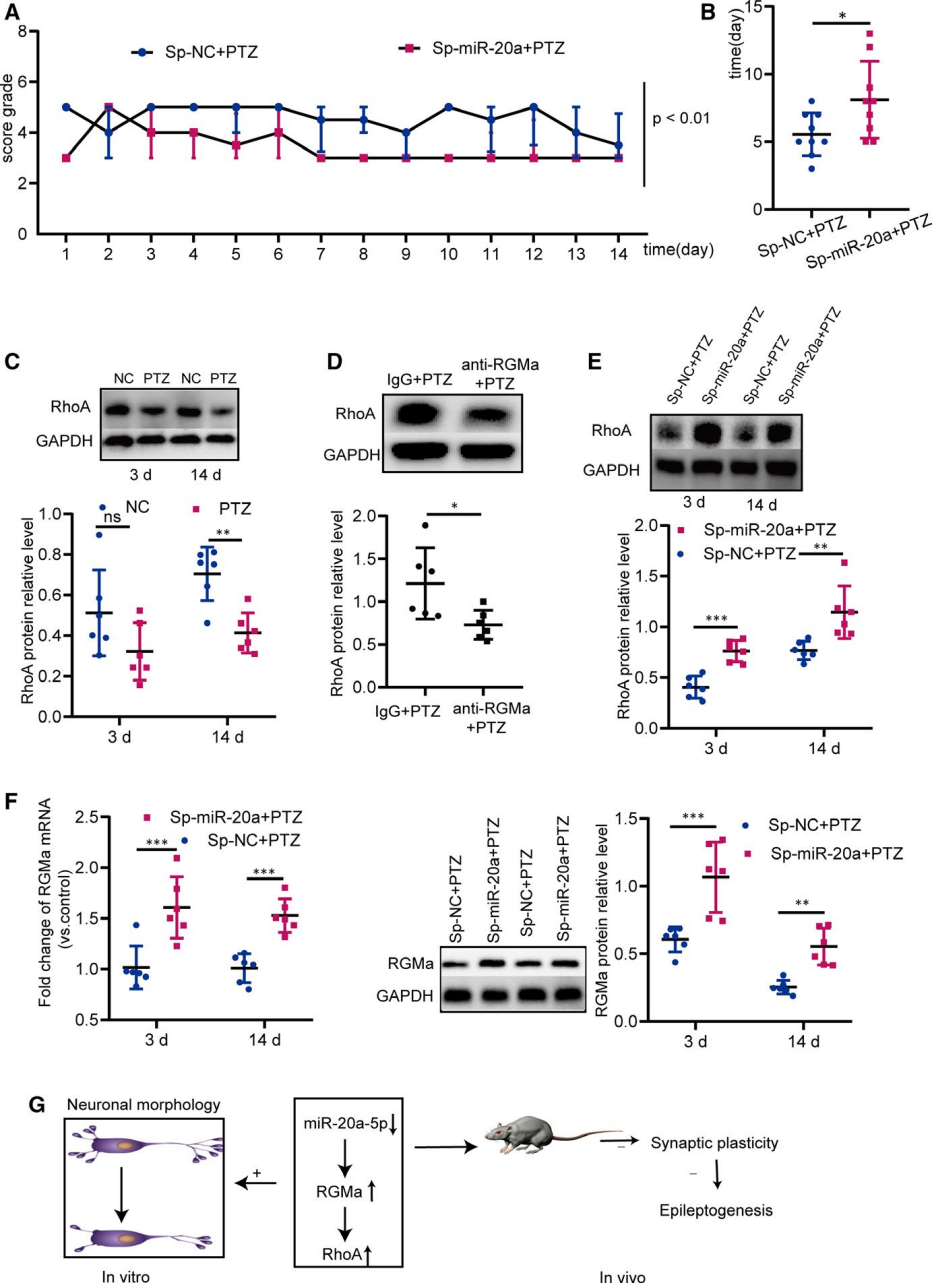
Antagonizing miR‐20a‐5p prevents epileptogenesis in the PTZ‐induced epilepsy rat model, and the miR‐20a‐5p‐RGMa‐RhoA pathway is involved in this process. A, Compared with rats in the Sp‐NC + PTZ group, antagonizing miR‐20a‐5p in Sp‐miR‐20a + PTZ rats decreased the seizure severity from 1 d to 14 d (n = 8, *P* < 0.01; log linear model). B, Down‐regulation of miR‐20a‐5p significantly delayed the occurrence of the first tonic‐clonic seizure (n = 8; two‐tailed t test). C, RhoA expression was distinctly lower in PTZ‐induced epilepsy rats than in control rats at 3 d and 14 d (n = 6; two‐way ANOVA, Sidak test). D, RhoA expression was significantly lower in anti‐RGMa + PTZ rats than in IgG + PTZ rats (n = 6; two‐tailed t test). E, Decreased miR‐20a‐5p increased RhoA expression in Sp‐miR‐20a + PTZ rats at 3 d and 14 d (n = 6; two‐way ANOVA, Sidak test). F, Sp‐miR‐20a + PTZ rats expressed higher RGMa mRNA and protein levels than Sp‐NC + PTZ rats at 3 d and 14 d (n = 6; two‐way ANOVA, Sidak test). G, Schematic diagram showing the mechanisms underlying miR‐20a‐5p‐RGMa‐RhoA pathway‐mediated synaptic plasticity in epileptogenesis: miR‐20a‐5p targeting RGMa regulates RhoA; down‐regulating miR‐20a‐5p reduces axonal growth and neuronal branching in vitro. Silencing miR‐20a‐5p prevents epileptogenesis through RGMa‐RhoA‐mediated synaptic plasticity in the PTZ‐induced epilepsy rat model. (^ns^ *P* ≥ 0.05, *P < 0.05, **P < 0.01, ***P < 0.001). All data represent the mean ± SD

Our previous analyses confirmed that miR‐20a‐5p down‐regulation suppresses synaptic plasticity in the PTZ‐induced epilepsy rat model. Past studies have indicated that synaptic plasticity promotes epileptogenesis.^12^ Next, we explored whether silencing miR‐20a‐5p affected epileptogenesis. The data showed that compared with those in the Sp‐NC + PTZ group, the seizure grades of rats in the Sp‐miR‐20a + PTZ group decreased significantly, and the occurrence of the first tonic‐clonic seizure was significantly delayed (Figure 7A,B). We further examined the miR‐20a‐5p‐RGMa‐RhoA pathway in PTZ‐induced rats. First, we examined whether RGMa regulates RhoA in epileptogenesis using the functional RGMa antibody. The hippocampal RhoA expression of PTZ rats was markedly lower than that of NC rats at 14 days (Figure 7C). When the biological functions of RGMa were interrupted by the functional RGMa antibody in PTZ rats, the level of RhoA was significantly lower in anti‐RGMa + PTZ rats than in IgG + PTZ rats (Figure 7D), indicating that RGMa regulates RhoA expression in epileptogenesis. Then, we further examined the miR‐20a‐5p‐RGMa‐RhoA pathway when miR‐20a‐5p was silenced by the miR‐20a sponge. Decreased RGMa levels were found during the kindling process (Figure 1C), and the RGMa protein and mRNA levels in hippocampal tissues at the injection sites in Sp‐miR‐20a + PTZ rats were higher than those in Sp‐NC + PTZ rats at 3 and 14 days, demonstrating that silencing miR‐20a‐5p increases RGMa expression (Figure 7F). The level of RhoA at the injection sites was distinctly increased in PTZ rats as demonstrated by significantly higher RhoA expression in the Sp‐miR‐20a + PTZ group than in the Sp‐NC + PTZ group at each time‐point (Figure 7E). These data indicated that the miR‐20a‐5p‐RGMa‐RhoA pathway is involved in synaptic plasticity‐regulated epileptogenesis. Taken together, these findings suggested that silencing miR‐20a‐5p prevents epileptogenesis through RGMa‐RhoA‐mediated synaptic plasticity in a PTZ‐induced epilepsy model.

## 4 DISCUSSION

The results of this study revealed the following: miR‐20a‐5p regulates RGMa and RGMa regulates RhoA—namely, miR‐20a‐5p, RGMa and RhoA coexist as a pathway; the miR‐20a‐5p‐RGMa‐RhoA pathway controls neuronal morphology in primary hippocampal neurons; silencing miR‐20a‐5p delays epileptogenesis through RGMa‐RhoA‐mediated synaptic plasticity but did not change MFS in the PTZ‐induced epilepsy rat model. These observations will be discussed in detail in the following sections.

Epilepsy is a chronic neurological disorder. Through the efforts of several generations of researchers, the mechanisms of epilepsy development have begun to be revealed. For example, RGMa prevents epileptogenesis and reduces seizure severity by inhibiting MFS in epilepsy animal models.^7^, ^8^, ^9^ However, the upstream regulators of RGMa in epileptogenesis have remained unknown. As upstream regulators of genes, miRNAs regulate multiple biological processes. To deeply understand RGMa functions and mechanisms in epileptogenesis, we inferred that miRNAs act as upstream regulators of RGMa in epileptogenesis. Based on three miRNA databases, miR‐106b‐5p, miR‐148b‐3p, miR‐152‐3p and miR‐20a‐5p were selected. These four miRNAs regulate tumorigenesis.^33^, ^34^, ^35^, ^36^ miR‐106b‐5p and miR‐20a‐5p also play important roles in central nervous system diseases.^30^, ^37^ Then, the levels of the four miRNAs were detected in PTZ‐induced epilepsy rats. The results showed that increased miR‐106b‐5p and miR‐20a‐5p levels led to an opposite trend of decreased RGMa levels. Using miR‐106b‐5p and miR‐20a‐5p inhibitors, we found that endogenous miR‐20a‐5p strongly affected RGMa protein expression. Moreover, we confirmed that miR‐20a‐5p directly binds to the RGMa 3'UTR and the regulatory relationship between miR‐20a‐5p and RGMa in PC12 cells and primary hippocampal neurons. Except for tumour‐related characteristics, miR‐20a‐5p also regulates neurite extension in the central nervous system.^30^, ^36^ Next, functional experiments were performed. Consistent with past studies, our results suggested that silencing miR‐20a‐5p inhibits axonal growth in primary hippocampal neurons. In addition, our present studies indicated that silencing miR‐20a‐5p also decreases neuronal branching in primary hippocampal neurons. Interestingly, these biological functions are mediated by RGMa. Existing mechanistic data^14^, ^21^, ^30^, ^38^ have indicated that RhoA may mediate miR‐20a‐5p/RGMa‐regulated changes in neuronal morphology. We confirmed the regulatory relationship between miR‐20a‐5p, RGMa and RhoA and that this pathway affects neuronal morphology. For the first time, our results showed that the miR‐20a‐5p‐RGMa‐RhoA pathway regulates the axonal growth and neuronal branching of primary hippocampal neurons.

Changes in axonal growth influence MFS; Changes in neuronal branching influence synaptic structural plasticity. Thus, the miR‐20a‐5p‐RGMa‐RhoA pathway may regulate MFS and synaptic plasticity in epileptogenesis. Based on the roles of MFS and synaptic plasticity in epileptogenesis, the miR‐20a‐5p‐RGMa‐RhoA pathway may modify epileptogenesis. RGMa, as the centre of this pathway, inhibits MFS in epileptogenesis according to a previous study.^7^ However, its roles in synaptic plasticity remain unknown. In the present studies, our data supported that RGMa suppresses MFS in epileptogenesis. Meanwhile, the in‐depth studies also indicated that RGMa inhibits synaptic plasticity in a PTZ‐induced epilepsy model, reinforcing evidence that the miR‐20a‐5p‐RGMa‐RhoA pathway may regulate epileptogenesis and deepening our understanding of the molecular mechanism of epileptogenesis. The roles of miRNAs in MFS remain unclear, except for silencing miR‐134 inhibiting MFS in a kainic acid‐induced epilepsy rat model.^29^ Regrettably, in our studies, in vivo miR‐20a‐5p silencing was not sufficient to reverse MFS, suggesting that MFS is independent of epileptogenesis in the context of miR‐20a‐5p silencing. The molecular mechanisms of MFS are complex. An increasing number of molecules regulating MFS have been identified.^39^, ^40^ Individual miRNAs are known to be able to regulate ~ 200 mRNAs each,^41^ and a single miRNA may regulate both mRNAs that promote and those that inhibit MFS. In the current study, we only focused on negative regulators of MFS, RGMa and RhoA. Facilitating factors may also have been increased by the decrease in miR‐20a‐5p expression. miRNAs potently regulate synaptic plasticity.^42^ However, whether miRNAs regulate synaptic plasticity in epileptogenesis is still unknown. In the current study, miR‐20a‐5p silencing decreased synaptic plasticity in the process of seizure genesis. This is the first miRNA identified to regulate synaptic plasticity in epileptogenesis. Meanwhile, this result also indicated that silencing miR‐20a‐5p has potential antiepileptogenic effects.

Current antiepileptic drugs do not show disease‐modifying properties. Therefore, we should aim to improve epileptic treatments and develop a new therapy to cure epilepsy. The regulatory relationships between epilepsy and miRNAs were first explored in 2010.^43^ miRNA expression profiles reveal that several miRNAs are dysregulated in patients with epilepsy.^24^, ^44^, ^45^, ^46^, ^47^, ^48^ Subsequently, in epilepsy animal models, neurologists have performed many miRNA functional and mechanistic studies in epileptogenesis. For example, miR‐134,^29^, ^49^ miR‐211,^50^ miR‐132 ^51^ and miR‐128^52^ strongly modify epileptogenesis. As a result, miRNAs have emerged as underlying targets for curing epilepsy.^23^ According to our above results, silencing miR‐20a‐5p reduces synaptic plasticity in epileptogenesis, and synaptic plasticity contributes to epileptogenesis ^12^; thus, silencing miR‐20a‐5p should delay epileptogenesis. Our studies confirm this inference as silencing miR‐20a‐5p was observed to reduce seizure severity and delay the occurrence of the first tonic‐clonic seizure in the PTZ‐induced epilepsy rat model. In addition, in 2012, genome‐wide microRNA profiling in patients with epilepsy revealed that miR‐20a‐5p expression is up‐regulated in the temporal lobe,^48^ suggesting that miR‐20a‐5p may play important roles in human epilepsy. Furthermore, we explored molecular mechanisms of miR‐20a‐5p‐mediated synaptic plasticity and further revealed molecular mechanisms of epileptogenesis. Many molecular mechanisms of synaptic plasticity have been described.^53^ Some studies have indicated that RhoA signalling impairs synaptic plasticity.^54^ In our study, silencing miR‐20a‐5p increased RhoA expression and decreased synaptic plasticity in the PTZ‐induced epilepsy model, which is consistent with previous studies. Moreover, our study also indicated that the RGMa‐RhoA pathway regulates synaptic plasticity in epileptogenesis. Finally, the roles of the miR‐20a‐5p‐RGMa‐RhoA pathway in synaptic plasticity were examined. miR‐20a‐5p was silenced with a sponge of miR‐20a in vivo, and silencing miR‐20a‐5p increased RGMa and RhoA expression, indicating that the miR‐20a‐5p‐RGMa‐RhoA pathway regulates synaptic plasticity in epileptogenesis. This is the first study to show that silencing of miR‐20a‐5p accompanied by increased RGMa and RhoA levels decreases synaptic plasticity in epileptogenesis, increasing the possibility that miR‐20a‐5 may be a target for curing epilepsy.

Collectively, a deeper understanding of the effects and mechanisms of RGMa and its downstream and upstream regulators in seizure genesis may help identify novel targets to arrest epileptogenesis and treat epilepsy. Here, we reveal that the miR‐20a‐5p‐RGMa‐RhoA pathway regulates axonal growth and neuronal branching in vitro and that silencing miR‐20a‐5p regulates epileptogenesis through RGMa‐RhoA‐mediated synaptic plasticity in a PTZ‐induced epilepsy model (Figure 7G). Thus, miR‐20a‐5p will be a new target for modifying epileptogenesis.

## CONFLICT OF INTEREST

The authors confirm that there are no conflicts of interest.

## AUTHOR CONTRIBUTION

**Yanyan Feng:** Conceptualization (equal); Data curation (lead); Formal analysis (lead); Funding acquisition (supporting); Methodology (lead); Software (lead); Writing‐original draft (lead); Writing‐review & editing (lead). **Chaojun Duan:** Conceptualization (lead); Methodology (equal); Resources (equal); Supervision (supporting). **Zhaohui Luo:** Methodology (equal); Supervision (equal). **Wenbiao Xiao:** Software (equal); Validation (equal). **Fafa Tian:** Funding acquisition (lead); Project administration (lead); Supervision (equal); Visualization (equal).

## Supporting information

Method S1

## Data Availability

The data that support the findings of this study are available from the corresponding author upon reasonable request.
