# Supplementary material for: Silencing miR‐20a‐5p inhibits axonal growth and neuronal branching and prevents epileptogenesis through RGMa‐RhoA‐mediated synaptic plasticity
Source: J Cell Mol Med. 2020 Aug 10;24(18):10573–88. doi: 10.1111/jcmm.15677 (PMC7521253; doi:10.1111/jcmm.15677)
Supplement: Supplementary file 1 — Method S1 [file JCMM-24-10573-s001.docx]

**Cell culture**

293T cells were acquired from the Chinese Academy of Sciences Cell Bank (Shanghai, China) and maintained in Dulbecco’s modified Eagle’s medium/nutrient mixture (Thermo Fisher, USA) containing 10% fetal calf serum. Cells were cultured at 37 °C in a humidified 5% CO_2_ atmosphere. The culture medium was replaced every 48 h until the cells were near confluent as observed under an inverted microscope (Leica, Germany).

Differentiated rat PC12 cells were purchased from the Chinese Academy of Sciences Cell Bank (Shanghai, China) and maintained with Roswell Park Memorial Institute 1640 medium (Thermo Fisher, USA) containing 10% fetal calf serum (Invitrogen, USA) at 37 °C in a 5% CO_2_ atmosphere. The culture medium was changed every 48 h until the cells were near confluent as observed under an inverted microscope.

Primary hippocampal neurons were prepared from neonatal rats (24 h postnatal). The hippocampi were dissected and dissociated into cells in Dulbecco’s modified Eagle’s medium/nutrient mixture F12 (Thermo Fisher, USA) including 2 mg/ml papain. The neurons were planted in plates coated with poly-d-lysine (0.1 mg/ml) at a density of 5×10^4^ neurons/cm^2^ and cultured in Dulbecco’s modified Eagle’s medium/nutrient mixture F12 containing 10% fetal calf serum at 37 °C in a 5% CO_2_ atmosphere. After 4-6 h, the medium was changed to Neurobasal A medium (Invitrogen, USA) containing 2% B27 (Invitrogen, USA), and 0.5 mM L-glutamine. Thereafter, the cultures were fed by replacing half of the medium every three days.

**Sample collection for RT-qPCR/ western blot and RNA/protein isolation**

For cell samples, stably expressing pre-miR-20a and miR-20a sponge PC12 cells and primary hippocampal neurons at 5 days in vitro (DIV) were gathered for quantitative real-time reverse transcription polymerase chain reaction (RT-qPCR)/western blot; normal PC12 cells and PC12 cells stably expressing pre-miR-20a or miR-20a sponge transiently transfected with Lipofectamine 2000 were collected 48 h later for RT-qPCR/western blot. The suitable TRIzol reagent (Life, USA)/RIPA buffer containing proteinase inhibitor was immediately added to the wells. The whole hippocampi in the anti-RGMa + PTZ and IgG groups were collected at 6 d, and the whole hippocampi in the NC group and PTZ groups and the hippocampal tissues of the injection sites in the Sp-miR-20a + PTZ and Sp-NC + PTZ groups were obtained at 3 d and 14 d. Then, the tissues were homogenized in TRIzol reagent/the RIPA: proteinase inhibitor mix. Total RNA was extracted using the TRIzol method according to the manufacturer’s instructions. The quality and concentration of the RNA were assessed using a spectrophotometer (BioTek, USA). For protein isolation, samples mixed with RIPA buffer and proteinase inhibitor were centrifuged for 5 min at 14 000 g to remove insoluble debris. Protein concentrations were measured with the bicinchoninic acid method. The extracted RNA and protein were stored at -80 °C until use.

**Luciferase/Renilla assay**

The direct binding of miR-20a-5p to the RGMa 3′UTR was evaluated by cloning wild-type and mutated RGMa 3′UTR fragments into the pmiR-RB-REPORT™ vector (RiboBio, China) downstream of Renilla luciferase. Then, the wild-type or mutant plasmid vector with control and miR-20a-5p mimics (RiboBio, China) were cotransfected into 293T cells with Lipofectamine 2000. This experiment was performed in triplicate. Forty-eight hours after the cells were incubated, luciferase activity was detected using a dual-luciferase reporter assay kit (Promega, USA). The luminescent signals were measured with luminescent light (Turner, USA). Relative fluorescence intensity = firefly fluorescence intensity/Renilla fluorescence intensity.

**RT-qPCR**

For miRNA expression analysis, 1000 ng of total RNA was retrotranscribed to cDNA using a universal cDNA synthesis kit (638313, Takara, USA) according to the manufacturer’s recommendation. For RGMa mRNA qPCR, 1000 ng of total RNA was reverse transcribed using a mRNA reverse transcription reagent kit (RR047A, Takara, USA) according to the manufacturer’s instructions. RT-qPCR was run on a StepOne Plus Real-Time PCR System (Applied Biosystems, USA) using SYBR Green Master Mix (RR820A, Takara, USA). All samples were run in duplicate. We normalized the expression levels of miRNAs and RGMa mRNA to those of U6 and glyceraldehyde-3-phosphate dehydrogenase (GAPDH), respectively. Relative fold changes in miRNA and RGMa mRNA expression were determined using the 2^−ΔΔCT^ method. The following primer pairs were used: miR-20a-5p: AAAGUGCUUAUAGUGCAGGUAG, miR-106b-5p: TAAAGTGCTGACAGTGCAGAT, miR-148b-3p: TCAGTGCATCACAGAACTTTGT, miR-152-3p: TCAGTGCATGACAGAACTTGG, RGMa forward: AAACACGGAGCCAACAGC and reverse: CCGCAGGCAGAGGTAGAG, GAPDH forward: CACGGCAAGTTCAACGGCACAGTCA and reverse: GTGAAGACGCCAGTAGACTCCACGAC.

**Western blot**

For protein analysis, western blotting was performed. Equal amounts of protein were separated through sodium dodecyl sulfate polyacrylamide gel electrophoresis. The protein samples were then transferred onto polyvinylidene fluoride membranes (PVDF) (Millipore, USA), which were then blocked for 90 min at room temperature in 5% nonfat milk diluted in TBST (Tris-buffered saline/Tween). The PVDF membranes were incubated with the following primary antibodies: rabbit anti-RGMa (1:4 000, ab169761, Abcam), rabbit anti-RhoA (1:1 000, ab1870027, Abcam), rabbit anti-postsynaptic density protein 95 (PSD-95) (1:1 000, ab18258, Abcam), rabbit anti-synaptophysin (SYP) (1:1 000, AF0257, Affinity), and rabbit anti-GAPDH (1:10 000, AC002, ABclonal) overnight at 4 °C. Afterward, the membrane was incubated at room temperature for 60 min with a solution of horseradish peroxidase-conjugated goat anti-rabbit (1:8 000, 074-1506, KPL) diluted in blocking buffer. The bands were visualized using an enhanced chemiluminescence detection system (ECL, Pierce, Thermo Fisher). Images were acquired with a Chemiluminescent Gel Imaging System (FluorChem FC3, ProteinSimple, USA), and densitometric analysis was performed with ImageJ software.

**Axonal length measurement and Sholl analysis**

To investigate the influences of silencing miR-20a-5p on axonal lengths and branches of primary hippocampal neurons, we imaged twenty neurons from each group using confocal laser scanning microscopy (Leica, Germany) equipped with appropriate excitation lasers and barrier filters. The longest neurites were measured using ImageJ software. Sholl analysis was executed with ImageJ software to assess neuronal branching. The area under the curve of the intersection numbers was calculated by GraphPad Prism 8 software.

**Slice preparation**

The brains subjected to Timm staining were cut into 30-µm coronal sections, and the hippocampal slices (120 µm section interval) were mounted on slides. The brains used for fluorescent scanning and immunofluorescence staining were coronally cut at 20 µm, and hippocampal slices (80 µm section interval) were mounted on slides. Finally, the sections were air-dried overnight and stored at -80 ℃ until use.

**Timm staining**

Slices were removed from storage at -80 ℃, dried in air, and stained in a solution containing 25 ml of 50% arabica gum, 2.1 ml of 2.55% citric acid, 12.5 ml of 5.67% hydroquinone, and 5 ml of 17% silver nitrate for 30-60 min at room temperature in the dark. The sections were then rinsed and dehydrated in alcohol, cleared in xylene, and mounted on slides with Permount. Three slices were selected randomly from each rat. Photomicrographs of the CA3 and DG region regions were captured from each animal with a microscope (Leica, Germany). The MFS values observed in the CA3 and DG regions were evaluated on a scale of 0-5, according to the Timm scale.

**Immunofluorescence staining**

Slices were removed from storage at -80 ℃ and dried in air. Slides were washed 3 times in PBS for 5 min. After the slides were blocked with 5% donkey serum in PBST (PBS Triton-X 100) and incubated at room temperature for 2 h, they were incubated with primary PSD-95 (1:400, ab18258, Abcam) and SYP (1:400, AF0257, Affinity) antibodies at 4 ℃ overnight. Next, at room temperature, the slices were incubated with secondary antibodies (1:500, 70-GAR5492, MultiScience) for 2 h. Then, nuclei were stained with Hoechst 33342 for 5 min. Finally, the sections were mounted with 50% glycerin. Three slices were selected randomly from each rat. Photomicrographs were captured for each animal with a microscope. The mean optical density values of the areas with positive immunolabeling were measured using ImageJ software. All assessments were performed in a randomized, blinded manner.
